# The impact of breathing route on pulmonary and sensory responses to ozone exposure during exercise in adults

**DOI:** 10.14814/phy2.71071

**Published:** 2026-08-24

**Authors:** André C. Silveira, Aidan K. Comeau, Matthew G. Skirrow, Jonah Brindley, Simon Boulianne, Christopher Hansen‐Barkun, Jem I. Arnold, Romulo Bertuzzi, Michael S. Koehle

**Affiliations:** ^1^ School of Kinesiology University of British Columbia Vancouver British Columbia Canada; ^2^ Endurance Performance Research Group (GEDAE‐USP), School of Physical Education and Sport University of São Paulo São Paulo SP Brazil; ^3^ Faculty of Medicine and Health Sciences McGill University Montréal Québec Canada; ^4^ Division of Sport & Exercise Medicine University of British Columbia Vancouver British Columbia Canada; ^5^ Laboratoire Motricité Humaine Expertise Sport Santé Université Côte d'Azur Nice France

**Keywords:** air pollution, dyspnea, exercise, oronasal, ozone

## Abstract

Ozone (O_3_) is a photochemical air pollutant known to reduce lung function and induce respiratory symptoms. This study aimed to determine whether breathing route influences O_3_‐induced lung function changes during exercise. Thirteen recreationally active participants (6 females, V̇O_2_peak = 45.8 ± 5.3 mL/kg/min), with no history of cardiovascular or pulmonary disease or exercise‐induced bronchoconstriction, completed a double‐blind crossover study with four experimental visits. Participants performed 30 min of low‐intensity cycling (90% of power at the first ventilatory threshold) while breathing exclusively nasally or orally under room air or 0.40 ppm O_3_. Spirometry was assessed before and after each trial, and dyspnea (Multidimensional Dyspnea Profile) every 5 min during exercise. O_3_ exposure impaired pulmonary function, reducing FEV_1_, FVC, PEF, FEF25–75, and FEV_1_/FVC (all *p* < 0.05). Regardless of environmental condition, oral breathing produced modestly greater post‐exercise reductions in FEV_1_, PEF, FEF25–75, and FEV_1_/FVC than nasal breathing, but no breathing route × environment interaction was observed. Nasal breathing was associated with greater breathing effort and air hunger (*p* < 0.05). Thus, O_3_ exposure impaired pulmonary function and increased dyspnea regardless of breathing route. Although nasal breathing slightly attenuated overall post‐exercise lung function decline, it did not protect against O_3_‐induced pulmonary impairment and increased breathing discomfort during exercise.

## Graphical abstract

Breathing routes elicited similar responses to O_3_ following submaximal exercise.

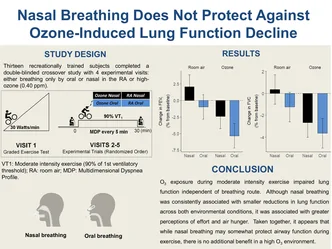

## 1 INTRODUCTION

Ground‐level ozone (O_3_) is a secondary air pollutant produced when ultraviolet light interacts with nitrogen dioxide (NO_2_) and non‐methane hydrocarbons (NMHC) (World Health Organization., 2021). When inhaled, O_3_ can react with components of the airway lining fluid, generating reactive oxygen species that promote epithelial injury, airway inflammation and activate sensory afferents, causing mainly chest discomfort, coughing, shortness of breath and throat irritation (Arsalane et al., 1995; Schelegle et al., 2007). These harmful effects appear to be more pronounced in people with chronic respiratory diseases such as asthma (Jarjour et al., 2013).

Additionally, these deleterious effects may be further exacerbated when O_3_ exposure occurs during exercise, as the exercise‐induced increase in minute ventilation ($\dot{V}$_E_) increases the inhaled dose of O_3_ (Harris et al., 2024). Although endurance exercise is well‐established as an important strategy for improving cardiorespiratory health (World Health Organization, 2010), exposure to even relatively low O_3_ concentrations (0.12 ppm) during 60 min of continuous cycling exercise has been shown to reduce forced expiratory volume in 1 s (FEV_1_) by 5.6% (Gong et al., 1986). Similarly, Balmes et al. (1996) reported reductions in FEV_1_ (−7.0%) and forced vital capacity (FVC) (−6.8%) following 4 h of exercise during exposure to 0.20 ppm O_3_ compared with filtered air. In the same study, the authors also demonstrated an inter‐individual variability in the pulmonary response to O_3_, with some individuals being more sensitive to O_3_ exposure than others, marked by greater reductions in lung function (FEV_1_: −36.0%; FVC: −28.0%).

In active individuals, nasal breathing is a strategy that might play a protective role in mitigating the impact of respiratory irritants (Hostetter et al., 2016). Nasal breathing has been shown to filter out both particles and gases, such as fine particulate matter (PM_2.5_) and O_3_, respectively (Oneda et al., 2026; Santiago et al., 2001; Sawyer et al., 2007). Nasal breathing may exert a protective effect during O_3_ exposure because the gas undergoes rapid chemical reactions within the nasal mucous layer, where it interacts with locally available substrates such as albumin, polyunsaturated fatty acids, and low‐molecular‐weight antioxidants in the upper airway (Santiago et al., 2001). These reactions contribute to scrubbing of inhaled O_3_ before it reaches the lower airways. Consistent with this mechanism, Kabel et al. (1994), demonstrated that approximately 80% of inhaled O_3_ was absorbed within the upper airways during nasal breathing, whereas only about 50% was removed during oral breathing. However, this potential protective mechanism may become less efficient during exercise, since most humans transition from nasal to oronasal breathing (i.e., simultaneously breathing through both nasal and oral route) with increasing exercise intensity (Fregosi & Lansing, 1995; Lacomb et al., 2017; Niinimaa et al., 1980). Saibene et al. (1978) demonstrated that this transition is not driven exclusively by physiological factors (i.e., $\dot{V}$_E_, nasal pressure and hypoventilation), but may also be influenced by psychological factors that mediate responses to the increased work of breathing. The authors reported that approximately 70% of participants believed that nasal breathing offers advantages during exercise, suggesting that such perceptions may influences the transition from nasal to oronasal breathing despite increasing ventilatory demands. In addition, the hypoventilation (and resultant hypoxemia) and the sensation of “air hunger” associated with nasal‐only breathing may increase chemoreceptor activity and, in turn, accelerate the transition from nasal to oronasal breathing (Illidi et al., 2023).

Oronasal breathing reduces the filtering advantage provided by the nose (Lizal et al., 2020). Therefore, breathing route may be a critical factor influencing O_3_‐induced health responses during exercise. Previous studies have provided important insights into the factors governing the transition from nasal to oronasal breathing (Saibene et al., 1978), and the effects of different breathing routes on lung function during O_3_ exposure (Hynes et al., 1988). However, these studies did not adequately quantify relative exercise intensity, which may influence breathing pattern to a greater extent than absolute exercise intensity (Hynes et al., 1988; Saibene et al., 1978) or lacked a clean air control condition (Hynes et al., 1988). Thus, a comparison of nasal and oronasal breathing routes under controlled conditions and at a controlled intensity was warranted. If nasal breathing were more protective than oral breathing, it could represent a simple and effective strategy to mitigate the harmful effects of exercising in polluted environments.

The aim of this study was to investigate the influence of breathing route (i.e., exclusive nasal vs. oral breathing) on pulmonary function responses and subjective experience of breathing discomfort (i.e., dyspnea) under during exercise O_3_ exposure. It was hypothesized that during exercise performed under higher O_3_ concentration, the nasal breathing may scrub O_3_, thereby contributing to protective effects on lung function.

## 2 MATERIALS AND METHODS

### 2.1 Experimental design

The sample size was estimated to be 12 participants with G*Power software (version 3.1.9.2) using data from previous investigations that analyzed the effect of oral vs. nasal‐only on oxygen consumption ($\dot{V}$ O_2_) breathing during exercise corresponding to 80% of $\dot{V}$O_2max_ (Lacomb et al., 2017). Participants were recruited through advertisements on public advertising boards around the university common areas and the recruiting website at the School of Kinesiology. Thirteen recreationally active subjects (7 male and 6 female) completed a double‐blinded crossover study comprising five visits. A minimum of 48 h and maximum of 7 days were permitted between each visit.

During the first session, participants were familiarized with the study protocol and provided written consent. They were then fitted with a mask and performed an incremental ramp test on a cycle ergometer to assess the ventilatory thresholds, peak oxygen uptake ($\dot{V}$ O_2peak_) and peak power output (PPO). The first ventilatory threshold was determined using the V‐slope method which identifies the breakpoint in the ventilatory response that corresponds to the onset of blood lactate accumulation above resting levels (Beaver et al., 1986; Keir et al., 2022). This assessment was used to confirm that the participants were classified as recreationally active (De Pauw et al., 2013; Decroix et al., 2016) and to determine the exercise intensity in the experimental trials.

During visits 2 to 5, the participants performed exercise at a low intensity (i.e., 90% of the power output corresponding to first ventilatory threshold) for 30 min. During these visits, the participants were exposed to either room air or O_3_‐polluted air using one of two respiratory routes: oral‐only (breathing exclusively through the mouth) or nasal‐only breathing (breathing exclusively through the nose). All visits were separated by 48 h to minimize carryover effects from previous exposure to O_3_. All tests were performed under consistent environmental conditions, temperature (23.7°C ± 0.38°C), humidity (32.9% ± 6.42%) and atmospheric pressure (755.0 ± 5.60 mmHg). Written informed consent was obtained from all participants prior to data collection. The study was approved by the University of British Columbia Clinical Research Ethics Board (H24‐01765) in accord with the Declaration of Helsinki.

### 2.2 Participants

The inclusion criteria for the study mandated that all participants were between the ages of 19 and 50 years old, non‐smokers, and able to safely perform a maximal exercise test. Individuals were not included if they had musculoskeletal injuries in the lower limbs that could interfere with or preclude cycling, were diagnosed with cardiorespiratory diseases (e.g., asthma) or vascular diseases that could impact performance. Participants were not previously adapted to nasal breathing during exercise.

### 2.3 Experimental setup

The experimental visits comprised four cycling bouts; (O_3_ exposure: oral or nasal‐only breathing either room air: oral or nasal‐only breathing) performed in a randomized, double‐blinded fashion. The participants were instructed to maintain the same self‐selected cadence throughout each trial (between 60 and 100 rpm). During exercise, participants were instrumented with a face mask connected to a non‐rebreathing valve (7450 Series V2 Mask, Hans Rudolf, Kansas, USA). During oral‐only breathing, the participants' nose was sealed using a low‐profile nose clip (Liquid Comfort Nose Clip, Speedo, Nottingham, UK) secured with medical tape. The nose clip was used during the oral‐only breathing to ensure exclusive oral inhalation and maximize the contrast between breathing conditions. This approach was chosen because, at the exercise intensity employed (~47% of PPO), nasal breathing could remain the predominant route of inspiration during unrestricted breathing, potentially reducing the distinction between oral and nasal breathing conditions. During the nasal‐only breathing route, participants wore medical tape over the mouth instead of the nose clip.

A 2‐min resting baseline was recorded immediately before exercise in both experimental conditions (Table S1). During this period, participants breathed room air, as O_3_ administration started only at the onset of exercise. Inhaled O_3_ concentrations were standardized to 0.40 ppm via a custom feedback control system connected to an air‐fed O_3_ generator (ACT5000, Mellifiq, Hägersten, Sweden). Since the O_3_ generator was supplied with ambient room air, the inspired gas mixture was expected to have the same temperature and humidity as room air. Inspired air was blended within a large enclosure before being delivered to the participant via a hose connected to the inspiratory port of the non‐rebreathing valve. O_3_ concentration was continuously monitored using a calibrated 49iQ Ozone Analyzer (Thermo Scientific, Waltham, MA, USA).

The expired gases and flows were measured by a metabolic cart (TrueOne 2400, ParvoMedics Inc., UT, USA) with heart rate (HR) measured with a paired Polar H10 HR transmitter (Polar Electro Oy, Kempele, Finland). End‐tidal CO_2_ (P_et_CO_2_) was derived from gas continuously sampled at the mouth (VacuMED CO_2_ Precision Gas Analyzer, Ventura, CA, USA) and recorded using an analog data acquisition system (PowerLab 16/35, 2014, ADInstruments Pty Ltd., Australia). Data were recorded using LabChart8 software for Windows (ADInstruments Pty Ltd., Australia).

Every 5 min during exercise, the participants were asked six questions assessing their subjective sensory and affective measures of dyspnea using the Multidimensional Dyspnea Profile (MDP) (Meek et al., 2012). Simultaneous ratings of perceived exertion using the Borg Scale were also recorded (6–20) (Borg, 1990).

### 2.4 Lung function

Participants performed spirometry before, immediately post, 15 min post, and 30 min post cycling exercise. Pulmonary function testing was performed using a calibrated MiniSpir spirometer (Rome, Italy) following the American Thoracic Society guidelines (Graham et al., 2019). This maneuver allowed for the calculation of FVC, FEV_1_, PEF, FEF_25‐75_ and the ratio FEV_1_/FVC. Trials were repeated at least three times until the two largest FEV_1_ and FVC values differed by ≤0.150 L.

### 2.5 Statistical analysis

Normality of data distribution was assessed using the Shapiro–Wilk test. Student's *t*‐test was used to compare baseline descriptive characteristics between males and females. For metabolic data, lung function and dyspnea, we utilized a linear mixed‐effect model to estimate the change compared from baseline to post‐exercise under each condition as expressed as a percent change from baseline. Fixed effects were breathing route (nasal vs. oral breathing) and environment (0.40 ppm O_3_ vs. room air). For lung function analyses, an initial model included post‐exercise sample timepoint (i.e., immediately, 15 min, and 30 min post‐exercise). Since preliminary analyses showed no significant condition × timepoint interaction or differences in the intervention effects across the post‐exercise timepoints. Therefore, a reduced model using only the immediate post‐exercise measurements was compared with the full model using Akaike's Information Criterion (AIC) and Bayesian Information Criterion (BIC). As the reduced model consistently demonstrated lower AIC and BIC values across all lung function outcomes, only baseline and immediate post‐exercise measurements were included in the primary analyses. For dyspnea, time was included in the model (every 5 min during 30‐min cycling test). Random effects of participant were included for the intercept of the models. Pairwise post hoc tests of estimated marginal contrasts were performed using the *emmeans* R package for each level of the fixed effects. Degrees of freedom were estimated using Kenward‐Roger method and multiple comparisons were adjusted with the *Tukey* method for comparing families of four paired estimates. Alpha was set at *p* < 0.05. All statistical analyses were conducted in R version 2025.05.0 (R. Posit Software, PBC, Boston, MA).

## 3 RESULTS

### 3.1 Environmental conditions and characteristics of the participants

Table 1 presents the main characteristics of study participants.

**TABLE 1:** Baseline characteristics of participants.

	Average	Males	Females
	(*n* = 13)	(*n* = 7)	(*n* = 6)
Age (years)	23.6 ± 3.7	23.9 ± 5.1	23.3 ± 1.2
Height (cm)	176.4 ± 10.3	184.4 ± 5.2	166.5 ± 4.1
Body mass (kg)	72.5 ± 9.1	78.5 ± 7.6	65.6 ± 4.9
$\dot{V}$O_2peak_ (mL·kg^−1^·min^−1^)	45.8 ± 5.3	48.5 ± 3.4	42.6 ± 5.5
Absolute $\dot{V}$O_2peak_ (L)	3.3 ± 0.6	3.8 ± 0.4	2.8 ± 0.4
$\dot{V}$O_2_ GET (L)	2.0 ± 0.4	2.3 ± 0.3	1.7 ± 0.3
$\dot{V}$O_2_ RCP (L)	2.7 ± 0.5	3.1 ± 0.3	2.3 ± 0.4
Maximal heart rate (bpm)	185 ± 8.8	185 ± 10.9	181 ± 9.8
RER	1.2 ± 0.09	1.2 ± 0.09	1.2 ± 0.04
PPO (W)	285.8 ± 61.1	327.5 ± 43.9	237.1 ± 36.9
Target PPO (W)	135.5 ± 32.5	152.4 ± 19.4	115.7 ± 34.8

*Note*: Data are mean ± SD.

Abbreviations: GET, gas exchange threshold; PPO, peak power output; RCP, respiratory compensation point; $\dot{V}$O_2_peak, peak rate of maximal oxygen uptake.

### 3.2 Respiratory variables during 30‐min constant load cycling trial

Table 2 shows the estimated marginal means and 95% confidence intervals from the metabolic variables during the 30‐min trial at a constant workload (i.e., target PPO) cycling. The $\dot{V}$_E_/$\dot{V}$O_2_ was significantly lower in nasal‐only breathing during O_3_ compared to oral‐only breathing in O_3_ and room air conditions (Δ$\dot{V}$_E_/$\dot{V}$O_2_: −1.90, *p* = 0.016 and Δ$\dot{V}$_E_/$\dot{V}$O_2_: 1.90, *p* = 0.017, respectively). A similar difference was observed for $\dot{V}$_E_/$\dot{V}$CO_2_ (Δ$\dot{V}$_E_/$\dot{V}$CO_2_: −1.90, *p* = 0.012 and Δ$\dot{V}$_E_/$\dot{V}$CO_2_: 1.83, *p* = 0.014 respectively). $\dot{V}$_E_ was only significantly different in the comparison between nasal‐only and oral‐only during O_3_ exposure (Δ$\dot{V}$_E_: −5.11, *p* = 0.037). P_et_CO_2_ showed higher values during nasal‐only breathing during exercise in O_3_ compared to both oral‐only condition in O_3_ and room air (Δ P_et_CO_2_: −2.89, *p* = 0.012 and Δ P_et_CO_2_: 2.89, *p* = 0.012, respectively).

**TABLE 2:** Model prediction values of the respiratory and metabolic variables during 30 min of cycling exercise.

	Nasal		Oral	
	O_3_	Room	O_3_	Room
Absolute $\dot{V}$O_2_ (L·min^−1^)	1.8 (1.6, 2.1)	1.9 (1.7, 2.2)	1.9 (1.7, 2.1)	1.9 (1.6, 2.1)
Absolute $\dot{V}$CO_2_ (L·min^−1^)	1.6 (1.5, 1.9)	1.8 (1.5, 2.0)	1.8 (1.5, 2.0)	1.7 (1.5, 2.0)
$\dot{V}$_E_/$\dot{V}$O_2_	23.8 (21.8, 25.8)	24.2 (22.2, 26.1)	25.7 (23.7, 27.6)^a^	25.7 (23.7, 27.6)^a^
$\dot{V}$_E_/$\dot{V}$CO_2_	25.8 (23.8, 27.7)	26.3 (24.4, 28.3)	27.6 (25.7, 29.6)^a^	27.6 (25.7, 29.5)^a^
$\dot{V}$_E_ (L·min^−1^)	43.1 (36.6, 49.6)	45.7 (39.2, 52.2)	48.2 (41.7, 54.7)^a^	47.5 (41.0, 54.0)
$\dot{V}$_T_ (L)	1.9 (1.6, 2.3)	2.0 (1.7, 2.4)	2.0 (1.7, 2.3)	2.1 (1.8, 2.4)
ƒ_B_ (breaths/min)	23.4 (19.8, 26.9)	23.9 (20.4, 27.4)	25.2 (21.7, 28.7)	24.9 (21.3, 28.4)
HR (bpm)	146.0 (137.0, 156.0)	144.0 (135.0, 154.0)	140.0 (131.0, 149.0)^a^	145.0 (135.0, 154.0)
Inhaled dose (ppm L)	516.1 (458.6, 573.5)	3.6 (−53.8, 61.1)^b^	577.3 (519.8, 634.7)	7.53 (−52.1, 67.1)^b^
P_et_CO_2_ (mmHg)	44.7 (40.9, 48.5)	44.1 (40.3, 47.9)	41.8 (38.0, 45.6)^a^	41.8 (38.0, 45.6)^a^

*Note*: Values are presented as estimated marginal means differences and 95% confidence intervals.

Abbreviations: $\dot{V}$E, minute ventilation; $\dot{V}$_T_, volume tidal; Absolute $\dot{V}$CO_2_, carbon dioxide production; Absolute $\dot{V}$O_2_, oxygen uptake; ƒ_B_, breathing frequency; HR, heart rate; P_et_CO_2_, Partial Pressure of End‐Tidal CO_2_.

^a^ *p* < 0.05 vs. Nasal + O_3_.

^b^ *p* < 0.001 vs. Oral + O_3_ and Nasal + O_3_.

### 3.3 Lung function

**FIGURE 1:**
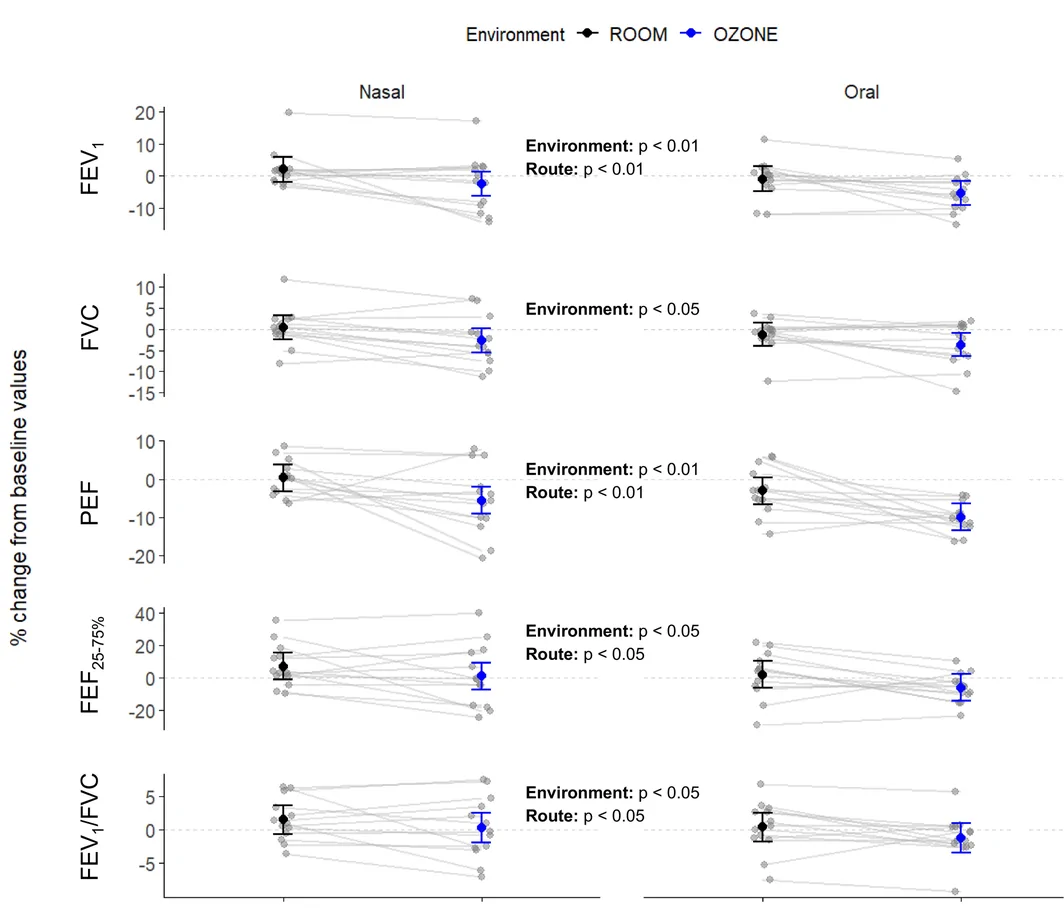
Estimated marginal means in lung function from baseline and spaghetti plot from individual values. FEV_1_, Forced expiratory volume in 1 s; FVC, Forced vital capacity; PEF, Peak expiratory flow; FEF_25‐75_, Forced expiratory flow from 25 to 75% of vital capacity. Values are presented as estimated marginal means and standard errors.

Figure 1 shows the estimated values from baseline immediately following 30 min of constant‐load cycling across breathing routes (oral vs. nasal) and environments (O_3_ vs. room air). Overall, there was a significant main effect of route for FEV_1_ (*p* = 0.014), PEF (*p* < 0.001), FEF_25–75_ (*p* = 0.023), and FEV_1_/FVC (*p* = 0.042), indicating slightly greater reduction in pulmonary function with the oral route compared to the nasal route in both environments.

In addition, there was a significant main effect of environment for FEV_1_ (*p* < 0.001), FVC (*p* = 0.003), PEF (*p* = 0.028), FEF_25–75_ (*p* = 0.012), and FEV_1_/FVC (*p* = 0.030), demonstrating that exposure to O_3_ led to greater impairments in lung function compared to room air. However, no significant interaction effects between breathing route and environment were observed for any of the pulmonary variables, suggesting that the effects of breathing route and O_3_ exposure were independent. The absolute estimated marginal means and their corresponding 95% confidence intervals for all spirometric variables are provided in Table S2.

### 3.4 Multidimensional dyspnea profile

**FIGURE 2:**
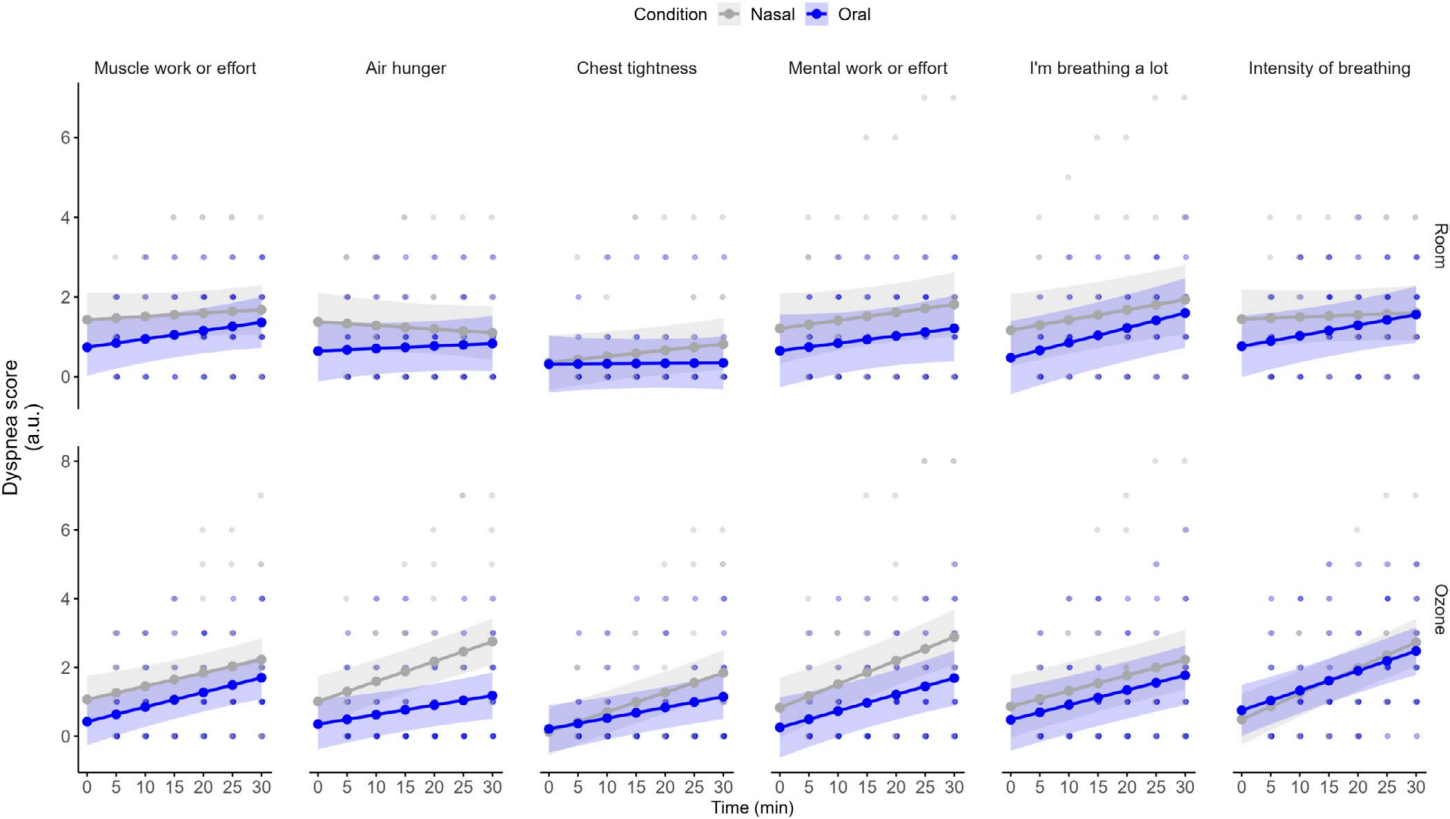
Estimated values for the multidimensional dyspnea profile throughout the 30‐min constant load cycling. Lines represent estimated marginal means (EMMs), shaded areas indicate the corresponding 95% confidence intervals, and dots plot individual values.

The multidimensional dyspnea profile (MDP) was evaluated every 5 min across all 30‐min exercise bouts. All questions (i.e., 1 to 6—Figure 2, Panels A to F) showed a significant main effect of time (all *p* ≤ 0.001), indicating that subjective scores of dyspnea symptoms increased over time during exercise.

Interestingly, a main effect of route was observed for “muscle work or effort” (*p* = 0.011), “air hunger” (*p* = 0.005), and “I'm breathing a lot” (*p* = 0.023), with higher scores in the nasal condition compared to oral. No main effect of environment was observed for most questions, except for “intensity of breathing”, which showed higher scores during O_3_ exposure compared to room air (*p* = 0.030). A significant interaction between time and route was observed only for “chest tightness” (*p* = 0.049). Additionally, a significant time × environment interaction was observed for “muscle work or effort” (*p* = 0.022), “air hunger” (*p* < 0.001), “chest tightness” (*p* < 0.001), “mental work or effort” (*p* = 0.010), and “intensity of breathing” (*p* < 0.001), indicating a greater progression of dyspnea symptoms over time during O_3_ exposure compared to room air.

## 4 DISCUSSION

The present study explored the differences in the respiratory responses between different breathing routes on lung function, metabolic variables and dyspnea symptoms during exercise performed in a high O_3_ environment and room air. The main findings of the present study were that lung function responses were consistently less impaired during the nasal route than the oral route, although no statistically significant differences were observed. However, comparison between environmental conditions showed that the nasal breathing route did not provide an additional protective effect. Specifically, O_3_ exposure impaired lung function compared with room air, but there was no significant interaction observed between breathing route and environment. However, the nasal route produced higher ratings of dyspnea than oral breathing. Taken together, these findings suggest that nasal‐only breathing may confer a modest bronchoprotective effect; however, this was not specific to O_3_ exposure.

### 4.1 Metabolic responses

Metabolic responses during the steady state exercise were influenced by breathing route than by environmental condition. Throughout exercise, $\dot{V}$_E_ /$\dot{V}$O_2_, $\dot{V}$_E_/$\dot{V}$CO_2_ and $\dot{V}$_E_ were higher during the oral than the nasal route. Similarly, (Lacomb et al., 2017) demonstrated a decrease in the $\dot{V}$_E_/$\dot{V}$O_2_, and $\dot{V}$_E_/$\dot{V}$CO_2_ during nasal‐only breathing during exercise. Contrary to these findings, (Hynes et al., 1988) showed no significant differences in $\dot{V}$_E_ in nasal breathing compared to oral breathing during 0.40 ppm O_3_ exposure and exercise. However, the previous data from (Hynes et al., 1988) are difficult to interpret since they did not adequately specify exercise intensity; the authors instead adjusted the ergometer to maintain the desired $\dot{V}$_E_ of 30 L/min. The experimental approach adopted by Hynes et al. (1988), in which $\dot{V}$_E_ was matched across breathing routes is useful for mechanistic investigations because it controls inhaled dose. Nevertheless, standardizing exercise intensity may better reflect real‐world exercise conditions, where individuals typically regulate exercise intensity based on external load (i.e., PO) rather than ventilation. Consequently, these approaches should be viewed as complementary. In the present study, PO was matched across conditions and routes, leading to a higher $\dot{V}$_E_ during oral‐only breathing. Although it did not result in a significant increase in inhaled dose, it was approximately 12% higher during oral compared with nasal breathing under O_3_ exposure. Nevertheless, this difference was not accompanied by differences in our primary outcomes. The observed decrease in $\dot{V}$_E_ during nasal breathing was likely related to a modest degree of hypoventilation (Illidi et al., 2023), as evidenced by the increase in P_et_CO_2_ during nasal breathing.

Our results also revealed that HR was higher in nasal breathing compared to oral breathing during O_3_ exposure. Given that nasal breathing is more efficient in terms of ventilatory equivalents, we did not expect differences in HR, as previously demonstrated in some studies comparing breathing routes during submaximal exercise (Lacomb et al., 2017; Yasuda et al., 1997). However, oral breathing provides less resistance to airflow through the pharyngeal passages. This reduced resistance could result in less mechanical work for the respiratory muscles during oral breathing, whereas the increased effort required for nasal breathing may have contributed to the higher HR. St Croix et al. (2000), demonstrated that increased inspiratory muscle work induced a reflex arising from fatiguing respiratory muscles, thereby augmenting sympathetic outflow, which could provide a potential physiological explanation for the increase in HR observed in our study. However, we did not directly assess respiratory muscle fatigue or sympathetic nerve activity; therefore, this mechanism should be considered speculative and warrants direct investigation in future studies. Although the higher P_et_CO_2_ observed during nasal‐only breathing suggests a modest degree of hypoventilation, the cardiovascular response to exercise reflects the integration of multiple mechanisms, including central command and feedback from group III/IV muscle afferents (Dominelli et al., 2021). Amann et al. (2009) demonstrated that using intrathecal fentanyl to attenuate group III/IV afferent feedback showed that elevated P_et_CO_2_ during a self‐paced exercise was accompanied by lower $\dot{V}$_E_, heart rate and arterial pressure. Thus, in the present study, the modest increase in P_et_CO_2_ alone is unlikely to be the only determinant of the cardiovascular response during exercise. Further studies should consider direct measurements of arterial blood gases, sympathetic activity, respiratory muscle work and respiratory muscle afferent feedback to better elucidate these interactions during nasal‐only breathing.

### 4.2 Lung function

The effect of O_3_ exposure during exercise on lung function has been previously demonstrated (Adams et al., 1981; Adams & Schelegle, 1983), however only one prior study has looked at the role of breathing route. Specifically, Hynes et al. (1988) compared nasal and oral breathing during combined 30‐min exercise and 0.40 ppm O_3_ exposure. O_3_‐induced reductions in FEV_1_ (−5.9%) and FVC (−4.5%) were observed; however, the magnitude of these changes was similar for both routes, indicating that the mode of breathing did not influence the extent of the pulmonary function impairment following O_3_ exposure in accordance with the present study. Of note, our inhaled dose was higher than reported by (Hynes et al., 1988) since they used exercise to elicit $\dot{V}$_E_ to 30 L/min for 30 min. The beneficial effect observed for FEV_1_, PEF, FEF_25‐75_, and FEV_1_/FVC ratio (but not FVC) in the present study indicates a general protective effect of nasal breathing to the increased airflow (and resultant cooling and drying) of exercise (Lacomb et al., 2017), and not a specific response to the O_3_‐induced changes which typically affect FVC as well as the airflow parameters (Adams & Schelegle, 1983).

### 4.3 Multidimensional dyspnea profile

As would be expected, we noted a main effect of environment on most of the questions as a result of the O_3_ exposure, which indicates that the environmental challenge was sufficient. With respect to breathing route, we observed that nasal breathing induced more pronounced discomfort related to “muscle work or effort of the breathing”, “air hunger”, and “breathing a lot” compared to oral. As mentioned above, these more pronounced symptoms are likely due to the potential hypoventilation associated with nasal breathing, especially in individuals who are not adapted to it (Illidi et al., 2023). This is further evidenced by the elevated P_et_CO_2_ during nasal‐only breathing. Consistent with these findings, (Ryan et al., 2022) demonstrated that experimentally induced hypercapnia during self‐paced exercise increased the perception of dyspnea. Collectively, these findings suggest that higher P_et_CO_2_ during nasal breathing was accompanied by greater respiratory drive and breathing discomfort. Therefore, although nasal breathing may provide a bronchoprotective effect during exercise regardless of the environmental condition, the accompanying increase in dyspnea may reduce adherence to this breathing strategy, particularly during prolonged exercise or among individuals unaccustomed to exclusive nasal breathing.

### 4.4 Limitations

This study was conducted with participants not previously adapted to nasal‐only breathing during exercise. This situation could have led to the greater scores in MDP when comparing nasal with oral‐only breathing. Moreover, the decrease in $\dot{V}$_E_ could also be related to a lack of adaptation of the participants with this breathing route. For instance, a case‐study of a triathlete adapted to nasal‐only breathing (Hostetter et al., 2016) did not show impairments in $\dot{V}$_E_ during a steady‐state test (i.e., 85% of the peak velocity during a maximal exercise test) and showed a decrease in air hunger sensation even with higher P_et_CO_2_ during exercise. Secondly, inflammatory marker assessment would have been advantageous since O_3_ exposure can increase the inflammatory response (Arsalane et al., 1995). Lastly, although no participant reported difficulty with nasal breathing, undiagnosed anatomical variations in the upper airway may have influenced the physiological responses observed during the nasal‐only breathing condition.

## 5 CONCLUSION

Our findings demonstrate that O_3_ exposure during moderate intensity exercise impaired lung function independent of breathing route. Although nasal breathing was consistently associated with smaller reductions in lung function across both environmental conditions, it was associated with greater perceptions of effort and air hunger. Taken together, our findings indicate that while nasal breathing may somewhat protect airway function during exercise, there is no additional benefit in a high O_3_ environment.

## AUTHOR CONTRIBUTIONS

**André C. Silveira:** Conceptualization; data curation; formal analysis; funding acquisition; investigation; methodology; validation; visualization. **Aidan K. Comeau:** Conceptualization; data curation; formal analysis; investigation; methodology. **Matthew G. Skirrow:** Data curation; methodology; validation. **Jonah Brindley:** Formal analysis; investigation. **Simon Boulianne:** Data curation; methodology. **Christopher Hansen‐Barkun:** Data curation; formal analysis. **Jem I. Arnold:** Conceptualization; data curation; formal analysis. **Romulo Bertuzzi:** Conceptualization; data curation; formal analysis; supervision; validation. **Michael S. Koehle:** Conceptualization; data curation; formal analysis; funding acquisition; project administration; supervision; visualization.

## FUNDING INFORMATION

This research was supported by São Paulo Research Foundation (FAPESP BEPE N°2023/17947‐2 and FAPESP N°2022/04960‐8), Coordination of Superior Level Staff Improvement‐Academic Excellence Program (CAPES‐PROEX‐Finance Code 001), and the Natural Sciences and Engineering Research Council of Canada (NSERC) under RGPIN‐2024‐04123 and RTI‐2021‐00091.

## CONFLICT OF INTEREST STATEMENT

The authors declare no conflicts of interest.

## ETHICS STATEMENT

The University of British Columbia's Clinical Research Ethics Board approved this study under the #H24‐01765.

## DISCLOSURE

Generative AI ChatGPT (OpenAI; GPT‐5.4 Thinking, 2026) was only used for minor modifications to R code. The authors directed the use of the AI technology and critically reviewed and tested all AI‐assisted code. Generative AI was not involved in the design of the study, analyses, interpretation of the results or the writing of this manuscript. Moreover, OpenAI's applicable Terms of Use, users retain ownership of their input and own the output, to the extent permitted by applicable law.

## Supporting information


**Table S1.** Model prediction values of the respiratory and metabolic variables during 2‐min baseline prior 30‐ min of cycling exercise.

**Table S2.** Model predicted percent change in pulmonary function outcomes from pre‐ to post‐ 30‐ min of cycling exercise.

## Data Availability

Data will be made available upon reasonable request.
