# Supplementary material for: The impact of breathing route on pulmonary and sensory responses to ozone exposure during exercise in adults
Source: Physiol Rep. 2026 Aug 24;14(16):e71071. doi: 10.14814/phy2.71071 (PMC13504239; doi:10.14814/phy2.71071)
Supplement: Supplementary file 1 [file PHY2-14-e71071-s001.docx]

**Supplementary Table 2.** Model predicted of absolute estimated marginal means and their corresponding 95% confidence intervals for all spirometric variables.

|  |  | **Nasal** | | **Oral** | |
| --- | --- | --- | --- | --- | --- |
|  | **Time Point** | **O_3_** | **Room** | **O_3_** | **Room** |
| FEV_1_ (L) | Pre | 4.40 (3.80,  5.03) | 4.53 (3.91,  5.20) | 4.33 (3.71,  5.00) | 4.50 (3.90,  5.13) |
|  | Post | 4.35 (3.72,  5.00) ** | 4.50 (3.85,  5.10) | 4.28 (3.65,  5.00) ** | 4.44 (3.82,  5.10) |
| FVC (L) | Pre | 5.59 (4.87,  6.32) | 5.69 (4.97,  6.41) | 5.52 (4.80,  6.24) | 5.69 (4.97, 6.41) |
|  | Post | 5.50 (4.77,  6.22) | 5.59 (4.87,  6.31) | 5.42 (4.70,  6.14) | 5.59 (4.87, 6.31) |
| PEF | Pre | 9.90 (8.11,  12.00) | 10.3 (8.52,  12.00) | 9.90 (8.10,  12.00) | 10.00 (8.23,  12.00) |
|  | Post *** | 9.43 (7.70,  11.20) ** | 9.84 (8.10,  12.00) | 9.42 (7.65,  11.20) ** | 9.55 (7.80,  11.30) |
| FEF_25-75_ | Pre | 3.90 (3.10,  4.71) | 4.14 (3.31,  5.00) | 3.90 (3.10,  4.71) | 4.10 (3.10,  4.92) |
|  | Post | 4.00 (3.20,  4.83) ** | 4.30 (3.43,  5.10) | 4.01 (3.20,  4.83) ** | 4.21 (3.40,  5.04) |
| FEV_1_:/FVC | Pre | 79.00 (74.00,  83.50) | 80.00 (75.00,  84.40) | 79.00 (74.00,  83.50) | 79.20 (74.40,  84.00) |
|  | Post | 79.00 (74.20,  84.00) | 80.00 (75.10,  85.00) | 79.00 (74.20,  84.00) | 79.40 (75.00,  84.20) |

FEV_1_: forced expiratory volume in 1 second; FVC: forced vital capacity; PEF: peak expiratory flow; FEF_25-75_: forced expiratory flow from 25 to 75% of vital capacity. Values are presented as estimated marginal means and 95% confidence intervals.

** p < 0.05 room vs. O_3_

*** p < 0.05 Pre vs. Post
