# Supplementary material for: The impact of breathing route on pulmonary and sensory responses to ozone exposure during exercise in adults
Source: Physiol Rep. 2026 Aug 24;14(16):e71071. doi: 10.14814/phy2.71071 (PMC13504239; doi:10.14814/phy2.71071)
Supplement: Supplementary file 2 [file PHY2-14-e71071-s002.docx]

**Supplementary Table 1.** Model prediction values of the respiratory and metabolic variables during 2-min baseline prior 30- minutes of cycling exercise.

|  |  | **Nasal** | | **Oral** | |
| --- | --- | --- | --- | --- | --- |
|  |  | **O_3_** | **Room** | **O_3_** | **Room** |
| Absolute $\dot{V}$O_2_  (L ∙ min^-1^) | | 0.34 (0.28,  0.41) | 0.38 (0.32,  0.45) | 0.40 (0.33,  0.46) | 0.38 (0.31,  0.44) |
| Absolute $\dot{V}$CO_2_  (L ∙ min^-1^) | | 0.31 (0.25,  0.36) | 0.34 (0.28,  0.40) | 0.35 (0.30, 0.40) | 0.33 (0.28,  0.39) |
| $\dot{V}$_E /_V̇O_2_ | | 33.0 (30.1,  35.9) | 34.0 (31.1,  36.9) | 32.7 (29.7,  35.6) | 32.1 (29.2, 35.0) |
| $\dot{V}$_E_ /V̇CO_2_ | | 36.7 (34.2,  39.2) | 37.8.0 (35.3,  40.4) | 36.2 (33.7,  38.7) | 35.6 (33.1, 38.1) |
| $\dot{V}$_E_ (L ∙ min^-1^) | | 11.7 (10.3,  13.2) | 12.6 (11.1,  14.0) | 12.6 (11.1,  14.1) | 11.7 (10.2,  13.2) |
| $\dot{V}$_T_ (L) | | 0.91 (0.67,  1.16) | 0.88 (0.64,  1.13) | 0.86 (0.61,  1.10) | 0.85 (0.61,  1.10) |
| ƒ_B_ (breaths/min) | | 15.7 (13.4,  18.0) | 16.3 (14.0,  18.6) | 15.8 (13.5,  18.1) | 14.6 (12.3,  16.9) |
| HR (bpm) | | 75.0 (61.4,  88.5) | 80.9.(67.3, 94.5) | 76.8 (63.2, 90.4) | 76.9 (63.3, 90.5) |

Absolute $\dot{V}$O_2_: oxygen uptake; Absolute $\dot{V}$CO_2_: carbon dioxide production; $\dot{V}$E: minute ventilation; $\dot{V}$_T_: volume tidal; ƒ_B_ breathing frequency; HR: heart rate. Values are presented as estimated marginal means differences and 95% confidence intervals.
